# A simplified protocol for DNA extraction from FTA cards for faecal microbiome studies

**DOI:** 10.1016/j.heliyon.2023.e12861

**Published:** 2023-01-07

**Authors:** Amanda Bolt Botnen, Mads Bjørn Bjørnsen, Antton Alberdi, M.Thomas P. Gilbert, Ostaizka Aizpurua

**Affiliations:** a Center for Evolutionary Hologenomics, The GLOBE Institute, University of Copenhagen, Denmark; b University Museum, NTNU, Trondheim, Norway

**Keywords:** 16S rRNA, Dry preservatives, Microbiota, Metagenomics, Metabarcoding, Longitudinal studies, PowerSoil, Cats

## Abstract

As metagenomic studies continue to increase in size and complexity, they are often required to incorporate data from geographically isolated locations or longitudinal time samples. This represents a technical challenge, given that many of the commonly used methods used for sample collection, storage, and DNA extraction are sensitive to differences related to the time, storage and chemistry involved. FTA cards have been previously proposed as a simple, reliable and cost-efficient method for the preservation of animal faecal microbiomes. In this study, we report a simplified extraction methodology for recovering microbiome DNA from faeces stored on FTA cards and compare its performance to a common alternative means of characterising such microbiomes; namely, immediate freezing of the faeces followed by DNA extraction using the Qiagen PowerSoil DNA isolation kit. Our results show that overall the application of our simplified DNA extraction methodology yields microbial community results that have higher diversity and an expanded core microbiome than that found using the PowerSoil methodology. This suggests that the FTA card extraction method presented here is a viable alternative for metagenomic studies using faecal material when traditional freeze-based storage methods are not feasible.

## Highlights

• FTA cards can aid gut microbiome sample collection from remote areas.
• We present a low-cost, simplified DNA isolation protocol.
• Higher diversity found with simple method compared to standard method.
• We conclude that this method can be a viable alternative where appropriate.

## 1 Introduction

The characterisation of animal gut microbial communities (microbiomes) is of increasing interest for both the basic and applied biological sciences [1]. In this regard, datasets are also increasing in size to explore variation within and between different populations or groups, as well as through time points (e.g. different developmental stages or ages). Due to ethical and technical constraints microbial characterisation of faecal material is often used as a proxy of the gut microbiome [2]. However, it is increasingly becoming apparent that the microbial community profiles recovered from such materials are very sensitive to both the conditions to preserve and store the sample [3,4].

The “gold standard” for the preservation of faecal material is rapid freezing at −80 °C [[5], [6], [7], [8]]. However, this is not always practical, especially considering the logistics of fieldwork and transportation. When rapid freezing cannot be assured, the use of liquid or dry preservatives are recommended [6,[8], [9], [10], [11], [12]]. Liquid preservatives such as 95% ethanol, DNA/RNA Shield (Zymo Research), and RNAlater (ThermoFisher), are the most commonly used preservatives in microbial studies. DNA/RNA Shield and RNAlater offer good but expensive alternatives when ethanol is impractical, such as when air travel is required. However, liquid preservatives add considerable weight and bulk and require cold chain management during transportation, which may be costly and challenging. Additionally, some liquid preservatives need to be removed from the faecal material before DNA extraction, which adds complexity and potential yield reduction to the extraction process, requiring more processing time and reagents or equipment. When cold-chain cannot be assured, dry preservatives such as faecal occult blood test cards (FOBT) or FTA cards (Whatman’s), are promising alternatives because i) they can easily be transported, ii) they can be stored at room temperature, and iii) they do not require a preservative buffer to be removed before extraction.

Despite the growing evidence that dry preservatives are a suitable means for preserving faecal material [6,8,10,13] their use in microbiome studies has remained limited. Nevertheless, examples of the successful uses of FTA cards for preserving faecal material for gut microbiomes exist for monkeys [14,15], birds [16], and humans [17]. It is possible that one reason for the relatively slow adoption of FTA cards relates to the low DNA yield when also using an extraction kit, although the low yield is also accompanied by a low amount of inhibitors [10]. However, in this regard, it is notable that the previously mentioned comparisons of the performance of microbiota preservatives against FTA cards all involved DNA extraction using the PowerSoil DNA Isolation kit (Qiagen) (or iterations upon this kit). We hypothesise that this may not be ideal for optimising microbial DNA recovery on FTA cards given the number of different steps involved in the process, all of which could lead to loss of the microbial DNA. To test this, we compared the performance of a simplified protocol for the extraction of DNA from FTA cards against the standard approach of directly freezing faeces upon collection coupled to DNA extraction with the PowerSoil method. Specifically, using cat faeces as a test substrate, we compared the resulting DNA yields, DNA fragment lengths, and microbial community diversity and composition.

## 2 Methods

When applied to faecal samples, the PowerSoil DNA Isolation kit protocol contains six key steps in the following order: preparation, lysis, inhibitor removal, binding DNA to spin filter, wash, and elution. In contrast, the alternate, simplified FTA card storage and DNA extraction method we explored reduces the number of steps and changes the order of operations. This is because according to the manufacturer, the FTA card itself lyses the cells upon contact, denatures proteins and protects the DNA from further degradation. Thus, the extraction protocol we employed consists only of resuspension of the DNA by adding the card to an elution buffer, followed by bead-based purification to isolate DNA strands from cell debris and inhibitors from the extracted sample. Given this simplicity, should the method prove effective, then it also not only is considerably cheaper but also is simpler to automate [[18], [19], [20]].

### 2.1 Sample preparation

Faecal samples were collected from seven cats at the “Kattens Værn” shelter, Brøndby, Denmark. The faecal material was immediately frozen on collection at −20 °C prior to use in the subsequent experiments. One experimental set, referred to hereafter as FTA + E, was subjected to FTA card (Whatman’s FTA Classic card) preservation at room temperature with desiccant packs, followed by the elution extraction method. The scats were thawed before placing a subsample onto a full circle of the FTA card with a sterile swab, then left to dry, as per manufacturer protocol, in a laminar flow hood. Blank cards were left open next to the drying cards to function as controls. The other group, referred to hereafter as F + PS, remained frozen until subjected to the DNeasy PowerSoil DNA Isolation kit (Qiagen) extraction protocol. Subsamples were prepared for extraction by placing 0.2 g faecal material into an Eppendorf tube. Because the amount subsampled for F + PS is considerably more than that added to FTA cards (approximately 0.01 g is used for the extraction), this is likely to have consequences regarding the total DNA extracted from each method. Both groups were subsampled in triplicate and blanks were included in all extraction rounds. After subsampling, samples were stored for a minimum of 24 h prior to extraction, at room temperature with desiccant packs in the case of FTA + E, or at −20 °C in the case of F + PS.

### 2.2 DNA extraction

DNA extractions were performed in batches to minimise cross-contamination between samples and methods. Each extraction batch consisted of one replicate for one of the two methods. For FTA card preserved material, a quarter-circle was cut with a scalpel from the sample area of each FTA card and placed in a Monarch spin column (NEB). The approximate weight of the cut card and faecal material combined was 0.01 g. To each tube, 175 μl of EB buffer was added before the tubes were incubated at 37 °C for 1 h. A 10-min centrifuge at 13,000×*g* removed the buffer and material from the card, and the subsequent supernatant was placed in a clean Eppendorf tube. A bead-based purification step was used to isolate the DNA from other cell debris following the AMPure bead protocol (Beckman Coulter, Brea, CA, USA) with a DNA: bead ratio of 1:1.4 with a final elution volume of 50 μl. A printable one-page protocol can be found in the supplementary material (supplementary material – printable protocol).

Extraction by the PowerSoil kit followed the manufacturer’s protocol. In brief, approximately 0.2 g of faecal material was added to a bead tube, along with solution C1, and vortexed vigorously. Each tube was then centrifuged at 10,000×*g* before adding solution C2 and incubating at 4 °C for 5 min and centrifuged again at 10,000×*g*. Solution C3 was then added followed by another 4 °C incubation for 5 min. DNA was then bound to a filter in a spin column along with solution C4, followed by two washes with solution C5, and a final elution step with 50 μl solution C6. Extraction blanks were included for each batch.

The concentrations of extracted DNA and average fragment lengths were estimated for each replicate by Fragment Analyzer (Agilent Technologies).

### 2.3 Bacterial metabarcoding and sequencing

Prior to metabarcoding, qPCR was used to screen all extracts at both 1:1 and 1:10 dilutions to test for the presence of any PCR inhibitors and estimate subsequent metabarcoding PCR dilutions. Two qPCR blanks and four blanks were included. The qPCR master mix per reaction consisted of 12.5 μl ddH_2_O, 2.5 μl 10× buffer, 2.5 μl MgCl_2_ 25 mM, 1.5 μl bovine serum albumin (BSA), 1 μl SYBR green, 0.5 μl 25 mM dNTP, and 0.5 μl AmpliTaq Gold, along with 2 μl 10 μM primer pair 341F/805R (forward: 5′-CCTACGGGNGGCWGCAG-3′ [21], reverse: 5′-GACTACHVGGGTATCTAATCC-3′ [22]) set to target the hypervariable V3 and V4 regions of the bacterial 16S rRNA gene, and 2 μl DNA. qPCR conditions were 95 °C for 10 min followed by 40 cycles of 95 °C for 15 s, 53 °C for 20 s, and 72 °C for 40 s. Post qPCR, gel electrophoresis was used to confirm correct amplicon length (∼450 base pairs).

Subsequently, metabarcoding was performed on each sample using unique nucleotide tags to allow multiplexing [23]. These unique tags consisted of 7–8 nucleotides and were added to the 5′ end of each primer (PCR master mix per reaction: 13.5 μl ddH_2_O, 2.5 μl 10× buffer, 2.5 μl MgCl_2_, 1.5 μl BSA, 0.5 μl dNTP 10 mM, 0.5 μl AmpliTaq Gold, 2 μl primer pair 341F/805R, and 2 μl DNA. PCR conditions: 95 °C for 10 min, 35 cycles of 95 °C for 15 s, 53 °C for 20 s, 72 °C for 40 s and a final extension at 72 °C for 10 min). Gel electrophoresis was used to confirm amplicon sizes after which the tagged amplicons were pooled into two pools before bead purifying (bead ratio 1:1, 30 μl elution buffer). Two pools were created for the library building, each containing up to 24 uniquely tagged sample replicates, bead purified and quantified by Qubit fluorometer (Invitrogen) (pool 1–242 ng/μl, pool 2–218 ng/μl). The unique tags allow for each of the three replicates per method per cat to be identified after sequencing. These pools were subsequently converted to Illumina compatible shotgun sequencing libraries with a modification of the Blunt-End Single Tube (BEST) library construction protocol [24], with 100 ng input DNA and 2 μl 50 mM adaptors. The two library pools were sequenced on an Illumina MiSeq platform (San Diego, CA, USA) (V3 Chemistry, 300bp paired), aiming for 5 million reads per pool, approximately 200,000 reads per PCR.

### 2.4 Bioinformatic data processing

Sequencing reads were demultiplexed using AdapterRemoval version 2.2.2 [25]. Since we applied an adapter ligation library building approach rather than PCR amplification, we identified primer locations using Cutadapt [26], and reads sequenced in the reverse-forward direction were reversed based on primer location to ensure unidirectionality of all sequences. Subsequent data processing was performed in R, version 4.0.2, and tools used are noted as *package::function*. The filtering and error models were done for each respective round of sequencing and primer directionality using DADA2 version 1.8 [27]. Forward and reverse reads were merged with a default overlap. We merged the four datasets and removed the chimeric sequences. The taxonomy assignment was performed using the Silva v138 taxonomy database [28,29]. Removal of contaminants was done using Decontam with the prevalence method and default settings [30]. Rarefaction curves were plotted using the function *ranacapa::ggrare* [31] and samples with a sequencing depth of less than 10,000 were discarded. To minimise the impact of low-abundance taxa, only ASVs with relative abundance higher than 0.01% were used in downstream analyses. A phylogenetic tree was generated by first aligning the ASVs using Clustal Omega [32] and subsequently building the tree using IQ-tree [33].

### 2.5 Statistical analysis

Concentration and fragment lengths were analysed using ANOVA. Diversity was calculated using Hill numbers [34,35] with *hilldiv::div_test* [36]. We calculated four alpha diversity metrics with combinations of three components of diversity: Richness, Evenness and Regularity [37]. The dR (Richness) measures the number of ASVs detected, without considering their relative abundances. The dRE (Richness + Evenness) measures the effective number of taxa, by accounting for the relative abundance of detected ASVs. The dRR (Richness + Regularity) measures the effective number of lineages, by accounting for the phylogenetic correlations of ASVs. The dRER (Richness + Evenness + Regularity) measures the effective number of lineages, by accounting for both the relative abundances and phylogenetic relationships among the ASVs. Diversity measures were analysed by linear mixed models, with cat number as the random effect (*lme4::glmer.nb* at dR, the rest *nlme::lme*).

To test the effect of the extraction method on bacterial community composition, we measured pairwise dissimilarities among all samples using the Jaccard-type overlap metric computed by *hill_div::pair_dis function* and performed a PERMANOVA analysis using *vegan::adonis* [31]*.* The aforementioned Jaccard-type dissimilarity matrix was used as response, *extraction method* was used as fixed explanatory factors and *Cat number* as strata. Core microbiota analysis was carried out using *microbiome::core* [38] with a 99% prevalence threshold. Differential abundance analysis was performed using a beta-binomial model controlling for cat number. The model was fit using *corncob::differentialTest* [39]. Statistical significance of differentially abundant ASVs determined through the parametric Wald test and using the default number of bootstrap simulations (n = 1000). Visualisations were performed using the ggplot2 package [40].

## 3 Results

### 3.1 DNA concentration, fragment length and rarefaction

**Fig. 1:**
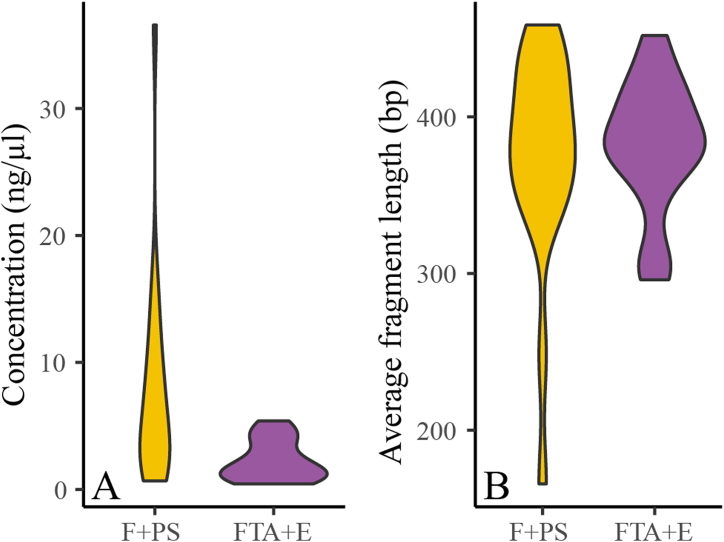
Comparison of (**A**) the concentration of DNA (ng/μl) and (**B**) the average fragment length (bp) from F + PS and FTA + E extracts. Replicates are combined and the mean concentration or fragment length is used.

After extraction, the DNA concentration was significantly lower in the FTA + E group 2.28 ± 1.60 ng/μl compared to F + PS 7.78 ± 8.26 ng/μl (p < 0.0001 and p(pooled) = 0.006, ANOVA, Fig. 1A), consistent with the fact that much less starting faecal material was used as input in the former (FTA + E had approximately 0.08 g while F + PS had 0.2 g input material). The average fragment length of the extracted DNA, prior to PCR amplification, was similar for both the FTA + E and F + PS methods (p = 0.627 and p(pooled) = 0.739, ANOVA, Fig. 1B).

**Fig. 2:**
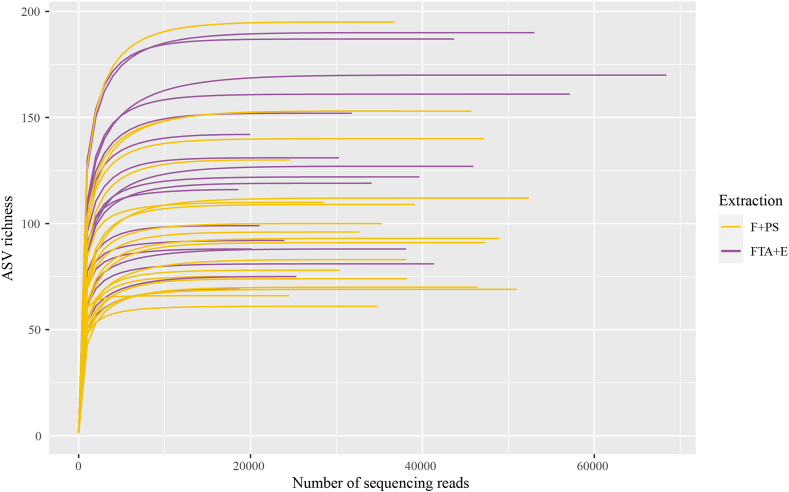
Rarefaction curves for each replicate showing that species richness is saturated for both extraction methods. Yellow indicates the F + PS method, while purple indicates the FTA + E method.

After processing the sequencing reads using DADA2, a total of 1151 ASV were identified across the 44 samples, including four blanks (FTA + E: 822 ASVs, n = 22, including 2 blanks, and F + PS: 745 ASVs, n = 22, including 2 blanks). In the FTA + E group, 12 contaminants were detected, but only one was present in a sample after filtering (ASV_183: Strectococcaceae). Within the F + PS group, four contaminants were detected of which one was present in the sample after filtering (ASV_464: Erysipelotrichaceae). After removing contaminants, a total of 1022 ASVs were kept (FTA + E: 765 ASV, n = 20 and F + PS: 695 ASVs, n = 20). After filtering low sequencing samples and low copy number ASVs, 774 ASVs remained across 39 samples in total (FTA + E: 603 ASVs, n = 19, and F + PS: 552 ASVs, n = 20). Finally, the ASVs that were not identified taxonomically as Bacteria (4 Eukaryotes) were filtered out and the output count table with 770 ASVs was used for downstream analyses. Rarefaction curves show species richness saturation for all samples of both methods (Fig. 2).

### 3.2 Diversity

**Fig. 3:**
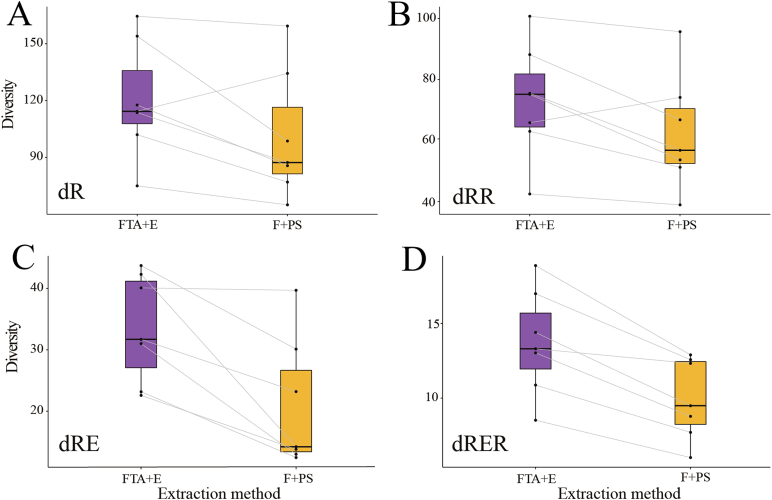
Diversity measure for FTA + E and F + PS methods at the ASV level. Grey lines connect the same individual between the two methods. (**A**) dR: number of taxa, (**B**) dRR: number of effective lineages, (**C**) dRE: number of taxa and their relative abundances, (**D**) dRER: takes into account the phylogenetic relationships. In all cases, diversity was higher for the FTA + E group.

Diversity measures were calculated based on Hill numbers, accounting for three aspects of diversity: richness, evenness and regularity [34,41]. For all four studied metrics, the mixed models revealed that alpha diversities of the gut microbial communities were significantly higher for the FTA + E group than F + PS (Fig. 3). Richness (dR), the number of taxa detected, i.e. alpha diversity, was higher for the FTA + E group than for the F + PS group (120.85 ± 38.52 and 102.85 ± 35.63 respectively, p = 0.01), explaining 8% of the variation. Richness and evenness (dRE), which incorporates the number of taxa and their relative abundances, was also higher for the FTA + E group than for the F + PS group (33.52 ± 12.09 and 21.34 ± 12.23 respectively, p < 0.001). The variation explained by the methodologies rose to 21% when relative abundances were accounted for. Richness and Regularity (dRR), which additionally accounts for the phylogenetic relationship between the taxa and therefore is a measure of the number of effective lineages, was higher for the FTA + E group than for the F + PS group (73.13 ± 22.88 and 63.64 ± 18.65 respectively, p = 0.03). The Richness, evenness and regularity metric (dRER) was also higher for the FTA + E group compared to the F + PS group (13.71 ± 4.44 and 10.17 ± 2.74 respectively, p < 0.001). When phylogenies were taken into account, the variance explained by the method was reduced to 6% (dRR) and 19% (dRER).

For each individual, dR ranged between 75.00 ± 8.49 and 164.50 ± 31.82 for the FTA + E group and 65.00 ± 5.66 to 159.33 ± 32.96 for the F + PS group. One cat contributed to the lowest diversity measures for both methods, and another cat contributed the highest diversity measure for both methods. dRE, indicating the effective number of taxa, spanned between 22.58 ± 15.84 and 43.74 ± 8.90 for the FTA + E group and 12.43 ± 1.03 and 39.70 ± 8.83 for the F + PS group. The effective number of lineages, dRR, spanned between 42.50 ± 5.83 and 100.74 ± 17.16 for the FTA + E group and 38.94 ± 4.63 to 95.68 ± 11.76 for the F + PS group. dRER, showing the effective number of lineages, ranged between 8.52 ± 0.42 and 18.87 ± 1.16 for the FTA + E group and 6.02 ± 0.10 to 12.89 ± 1.98 for the F + PS group.

### 3.3 Microbial compositional variation

**Fig. 4:**
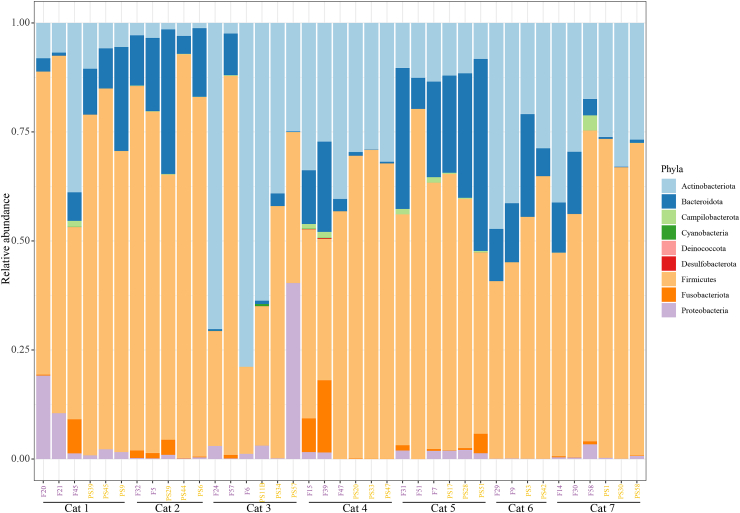
Barplot of the microbial composition at phylum level for each sample among the seven cats. F indicates the FTA + E method, while PS indicates the F + PS method.

At the phylum level, the microbial composition varied between replicates of both FTA + E and F + PS methods (Fig. 4). Eight phyla were found in both groups: Actinobacteriota, Bacteroidota, Campilobacterota, Cyanobacteria, Desulfobacterota, Firmicutes, Fusobacteriota, and Proteobacteria. The three most dominant phyla for both methods were Firmicutes (FTA + E: 57%, F + PS: 64%), Actinobacteriota (FTA + E: 28%, F + PS: 20%), Bacteroidota (FTA + E: 11%, F + PS: 11%). Although diversity was slightly higher in the FTA + E group, a Wilcoxon test indicated that there was no difference in composition at the phylum level between extraction groups of the same faecal sample, except for Campylobacterota, which although relatively rare overall in the dataset, was statistically significantly more abundant in the FTA + E group (W = 9.0, p = 0.027). Deinococcota were only found in the FTA + E sample of one cat.

**Fig. 5:**
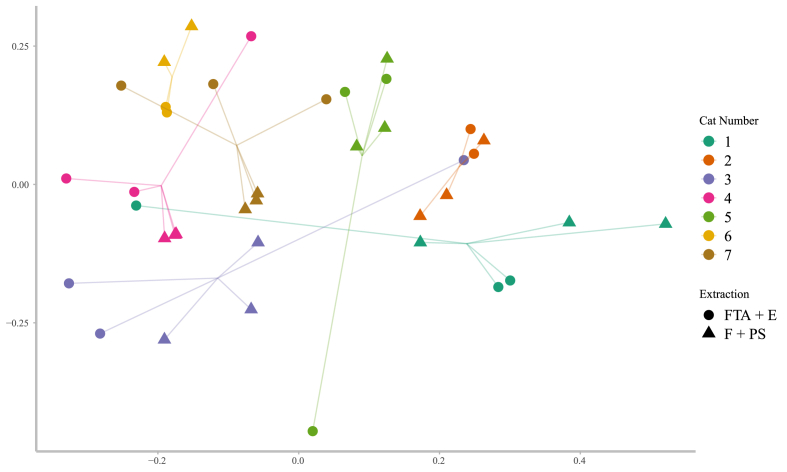
NMDS plot of compositional variation for each sample, taking into account the richness and evenness. Triangular shapes represent the FTA + E group, while circular shapes represent the F + PS group.

The level of homogeneity of dispersion within groups was analysed using vegan::betadisper and vegan::permutest functions [31]. Since this assumption was met with all computed metrics (p value > 0.05), we performed the PERMANOVA analysis based on pairwise distances calculated through the four diversity metrics. The PERMANOVA analysis showed that the variance explained by extraction method was low, although increasing as more complex metrics of diversity were used (dR: R^2^ = 3%, dRE: R^2^ = 3%, dRR: R^2^ = 6%, dRER: R^2^ = 8%, visualised for dRE in Fig. 5). However, the differential abundance was also not significantly different. Although there was variability among replicates, the coefficient of variation was not significantly different for the two methods (Wilcoxon test: dR: p = 0.16, dRE: p = 0.58, dRR: p = 0.11, dRER: p = 0.16).

### 3.4 Core microbiome

The core microbiota of both groups shared seven common genera: the Firmicutes (*Peptoclostridium Clostridium*, *Blautia*, and *Lachnoclostridium*), and the Actinobacteria (*Collinsella*, *Bifidobacterium* and *Slackia*). The FTA + E group also included 10 additional bacterial genera: the Firmicutes (*Catenibacterium*, *Holdemanella*, *Lactobacillus*, *Enterococcus*, *Peptococcus*, and *Subdoligranulum*), the Actinobacteria (*Olsenella* and *Libanicoccus*), the Bacteroidetes (*Prevotella*) and the Proteobacteria (*Escherichia/Shigella*).

## 4 Discussion

Faecal material from seven cats was divided into two groups: a first group involving preservation on FTA cards followed by DNA extraction using the simplified elution method presented here (FTA + E), and a second group in which DNA was extracted with the DNeasy PowerSoil Isolation Kit (F + PS). As such, each faecal sample from the seven cats underwent both methods in triplicate. We found that the DNA yield of the FTA + E group was lower than for the F + PS group, which was almost certainly due to the much lower amount of input faecal material initially used. Using more input runs the risk of clogging the filter, again limiting yield. Including the filter paper itself. Approximately 0.01 g of input material was used for each triplicate in the FTA + E method compared to 0.2 g with F + PS. Others have also noted that the low input is the cause of the low yield when using FTA cards, regardless of extraction method [10]. The bead purification step also results in sharp cut-offs in fragment length for the FTA + E group, which may be relevant for shotgun libraries but does not impact amplicon libraries.

While overall the sample effect was clearly the major driver of differences in the microbial profiles, as similarly reported elsewhere [42], we nevertheless observed a small, but significant difference in the microbial community diversity and core diversity profiles recovered with the tested methods. Despite the lower DNA yield, the FTA + E group showed a higher microbial diversity. Campylobacterota was the only phyla significantly influenced by the method, which may need to be kept in consideration by future users of the methods given it is a common inhabitant in the feline gut microbiome that has been reported to potentially cause enteritis in both pets and owners [43].

Further investigation into the additional core microbiome bacteria recovered in the FTA + E group reveals all represent gut bacteria, thus likely deriving from the faecal samples. However, the two methods recover slightly different core microbiomes. The differences seem not to be related to cell wall structure as the additional core microbiota recovered from the FTA cards are evenly distributed among gram positive and gram negative bacteria. Both Firmicutes and Actinobacteria are gram-positive, while Bacteroidetes and Proteobacteria are gram-negative. Additionally, the removal of PCR inhibitors may be much more efficient with a bead-based purification step incorporated in the FTA + E method compared to the inhibitor removal step in the DNeasy PowerSoil Kit, resulting in better amplification of the sample. However, efficient inhibitor removal is sometimes the aim of new interactions of a DNA isolation kit. After the completion of the laboratory work for this study, the manufacturer protocol of a newly released DNeasy PowerSoil Pro Kit (Qiagen) showed improved inhibitor removal and increased diversity compared to the PureLink Microbiome DNA Purification Kit (ThermoFisher). Continuous improvements to the DNA extraction kit, while beneficial for the singular study, can be a problem with long-term studies when results are no longer comparable due to differences in kit effectiveness. FTA cards, on the other hand, are routinely used in governmental programmes such as the dried blood spots for newborn screening of metabolic disorders [[44], [45], [46]]. This long-term, global application of FTA cards may make them more resistant to change over time. As noted previously, the overall lower concentration in FTA + E also reduces the amount of PCR inhibitors present.

Immediate freezing or 95% ethanol preservation, followed by the PowerSoil DNA Isolation Kit is the most commonly used preservation and extraction method for microbiome studies, including for the Earth Microbiome Project when the KingFisher robot is not available [47]. In situations where the “gold standard” preservation method is not feasible, alternative methods are needed. While all scats in this study were frozen before preparation for the two extraction methods, the purpose of the study is to offer an alternative extraction method when conditions do not allow for adequate freezing or timely transport. Unpublished data using fresh stool sample show similar results. The method presented here has practical benefits of using FTA cards as both preservative material and the simplified extraction protocol. FTA cards take up very little space and do not require freezing. This reduces the cost of both transport and long-term storage. Since cell lysis occurs on the card itself, the first step of the extraction protocol has already been completed. An extraction method, consisting of a simple elution and bead purification, is sufficient to isolate microbial DNA for microbiome community analysis. Separating the protocol from commercial extraction kits is beneficial for reproducibility years or decades later, especially relevant for longitudinal studies. The filter paper itself is unlikely to undergo significant changes due to its large-scale use in diagnostics and forensics. Due to the wide variety of large-scale uses of filter paper, automation methods are already in place [48]. The FTA card protocol described above uses only elution buffer, ethanol, and locally produced SPRI beads made with common generic reagents. One concern regarding FTA cards is that the cards are left open to dry, potentially exposing them to environmental and cross-contamination. However, negative controls that were left open next to the drying cards were all blank.

FTA cards offer an affordable, easy-to-use alternative method for microbiota preservation for metagenomics when the cold chain cannot be maintained or standardisation is required for long-term projects. FTA cards have previously been deemed suitable as a preservation method for microbiome studies yet have been rarely utilised. Utilisation of FTA cards may have been hindered by the use of a full DNA isolation kit reduced the DNA yield to below a useable threshold, or that samples are collected locally and therefore do not have the same challenges with cold chain management. However, FTA cards may be particularly relevant in cases where remote or long-term sampling or a large number of samples are required. The cost-effective and simple sampling, transport, and storage can aid citizen science projects, which dramatically increases the range of biologically interesting samples, and potentially reduces geographical sampling bias. Remote locations and cold chain management are not a risk to the samples, even in extreme cases where samples may be caught in customs for weeks or months. Automating much of the process may also reduce the risk of repetitive strain injury among laboratory workers, an important but often overlooked aspect of large studies.

Finally, we caveat that while our research is promising, we cannot rule out that the differences in microbial community between the two methods are the result of species-specific differences in stool condition and composition and are based on a small sample size. Future studies using mock communities are necessary to uncover these potential biases. Furthermore, this study looked only at the hypervariable V3–V4 region of the 16S rRNA gene. Shotgun sequencing from FTA cards may discover additional differences between the two methods analysed here.

## Author contribution statement

Amanda Bolt Botnen: Conceived and designed the experiments; Performed the experiments; Analyzed and interpreted the data; Contributed reagents, materials, analysis tools or data; Wrote the paper.

Mads Bjørn Bjørnsen: Performed the experiments; Contributed reagents, materials, analysis tools or data; Contributed reagents, materials, analysis tools or data.

Antton Alberdi, M Thomas P Gilbert: Conceived and designed the experiments; Analyzed and interpreted the data; Contributed reagents, materials, analysis tools or data; Wrote the paper.

Ostaizka Aizpurua: Analyzed and interpreted the data; Contributed reagents, materials, analysis tools or data; Wrote the paper.

## Funding statement

Professor M Thomas P Gilbert was supported by Villum Fonden [17417], Danmarks Grundforskningsfond [DNRF143].

## Data availability statement

Data will be made available on request.

## Declaration of interest’s statement

The authors declare no competing interests.
