# Supplementary figures and images for: A simplified protocol for DNA extraction from FTA cards for faecal microbiome studies
Source: Heliyon. 2023 Jan 7;9(1):e12861. doi: 10.1016/j.heliyon.2023.e12861 (PMC9868478; doi:10.1016/j.heliyon.2023.e12861)

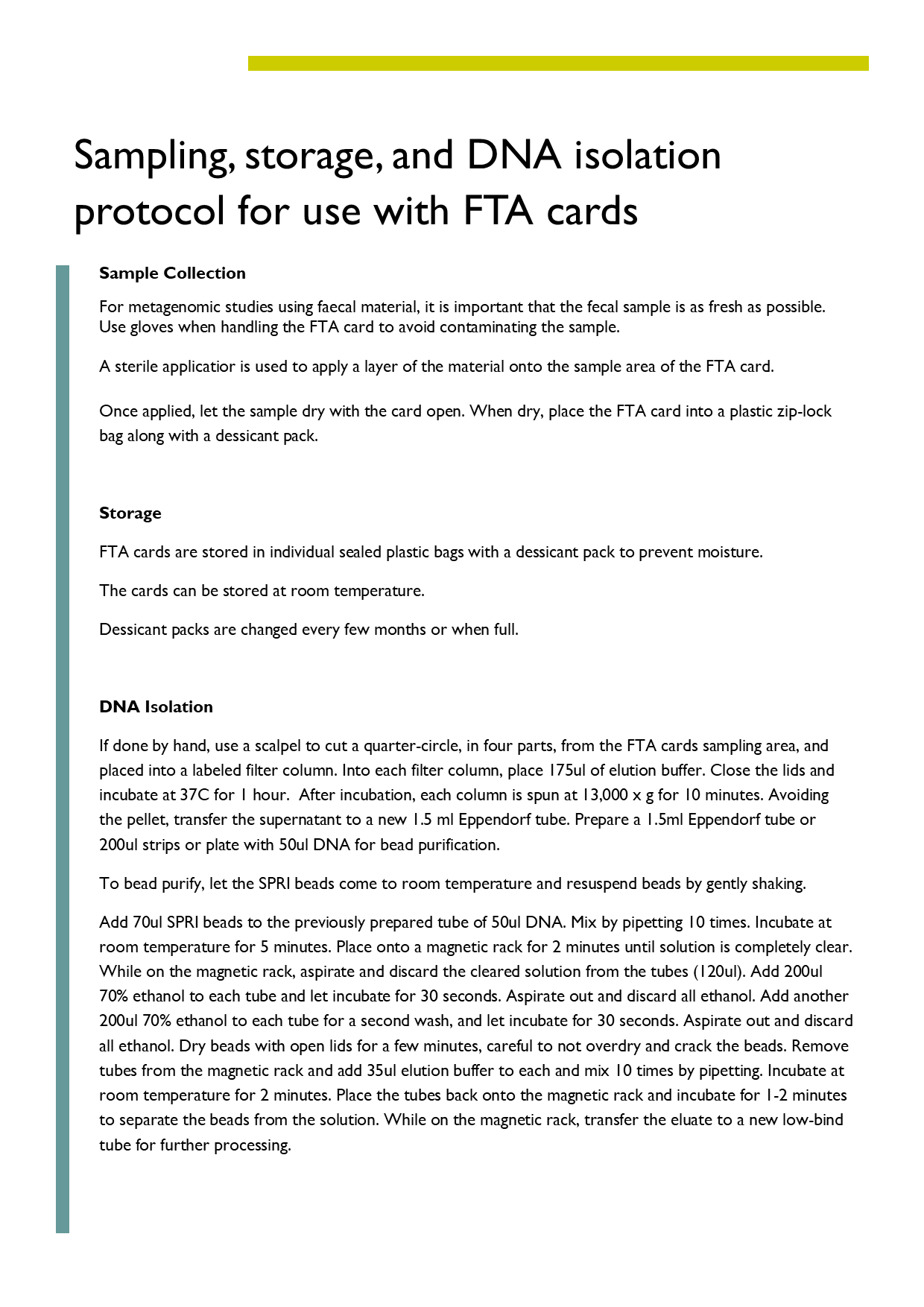

Supplement: Multimedia component 1 [file mmc1.docx]
